# Mammography Datasets for Neural Networks—Survey

**DOI:** 10.3390/jimaging9050095

**Published:** 2023-05-10

**Authors:** Adam Mračko, Lucia Vanovčanová, Ivan Cimrák

**Affiliations:** 1 Faculty of Management Science and Informatics, University of Žilina, 010 26 Žilina, Slovakia; 2 Research Centre, University of Žilina, 010 26 Žilina, Slovakia; 3 2nd Radiology Department, Faculty of Medicine, Comenius University in Bratislava, 813 72 Bratislava, Slovakia; 4 St. Elizabeth Cancer Institute, 812 50 Bratislava, Slovakia

**Keywords:** mammograms, open-access databases, deep neural networks, artificial intelligence, mammography, machine learning

## Abstract

Deep neural networks have gained popularity in the field of mammography. Data play an integral role in training these models, as training algorithms requires a large amount of data to capture the general relationship between the model’s input and output. Open-access databases are the most accessible source of mammography data for training neural networks. Our work focuses on conducting a comprehensive survey of mammography databases that contain images with defined abnormal areas of interest. The survey includes databases such as INbreast, the Curated Breast Imaging Subset of Digital Database for Screening Mammography (CBIS-DDSM), the OPTIMAM Medical Image Database (OMI-DB), and The Mammographic Image Analysis Society Digital Mammogram Database (MIAS). Additionally, we surveyed recent studies that have utilized these databases in conjunction with neural networks and the results they have achieved. From these databases, it is possible to obtain at least 3801 unique images with 4125 described findings from approximately 1842 patients. The number of patients with important findings can be increased to approximately 14,474, depending on the type of agreement with the OPTIMAM team. Furthermore, we provide a description of the annotation process for mammography images to enhance the understanding of the information gained from these datasets.

## 1. Introduction

Breast cancer is the most prevalent cancer type among females. According to the GCO [1], the incidence rate of the disease is 47.8 by ASR (age-standardized rate), which translates to almost 48 women per 100,000 per year. This rate is nearly three times higher than the second most prevalent cancer. Furthermore, breast cancer has the highest mortality rate, with a rate of 13.6 by ASR. As stated by the American Cancer Society [2], when breast cancer is detected early and is in the localized stage, the 5-year relative survival rate is 99%. The stage of the disease is the most important factor for treatment selection and prognosis prediction. Therefore, mammographic screening has been established as the best method for detecting breast cancer in its early stages in many countries.

The screening is periodically performed and consists of testing healthy individuals to identify those with cancers before any symptoms appear [3]. Women in the non-risk group undergo mammography every two years, while those with high risk undergo it annually. Several risk factors, such as sex (females), age, family history, hormone therapy, early menarche, late menopause, radiation exposure, etc., have been identified [4]. Screening results in the detection of many cases that require further examinations and treatment, creating a high time burden for medical personnel. Additionally, double reading [5] is applied, wherein two radiologists independently read the same mammograms to reduce errors and increase the quality of annotation. If both doctors agree that there is a suspicious finding in the breast, a tissue biopsy is necessary. However, many of these biopsies turn out to be benign breast lesions, accounting for 55% to 85% of cases in the USA [6]. Mammography is a breast imaging method that uses ionizing radiation (X-rays). In the older method, SFM (screen-film mammography), the mammogram is obtained by exposing the film to the radiation produced by an X-ray tube. The modern method, FFDM (full-field digital mammography or digital mammography), has replaced the film with a digital receptor that converts the residual radiation into an electrical signal. FFDM is the only method approved for mammographic screening performance. Transition to FFDM has revealed that it performs as well as SFM [7]. According to some studies, FFDM is even more accurate in women under 50 years old [8] and in the detection rate of interval cancer [9].

The high time burden of mammographic screening creates opportunities for computer diagnostic assistance tools. If these tools are able to achieve results similar to or better than radiologists, then double reading could potentially be conducted with the assistance of a tool and one radiologist. Recently, the most popular methods applied to analyze visual imagery have been deep convolutional neural networks (DCNNs). However, training and testing DCNNs require large datasets, including labeled mammography images. Open databases provide valuable resources for these images. Even if a model is trained on a private dataset, open databases can still be used for additional validation of the final model.

## 2. Mammography Assessment Process

**Figure 1:**
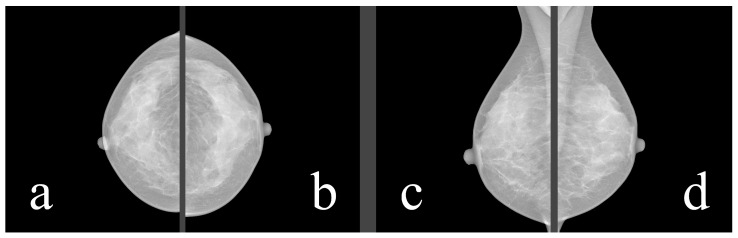
Four standard views: (**a**) right CC, (**b**) left CC, (**c**) right MLO, (**d**) left MLO. Source: [11].

**Figure 2:**
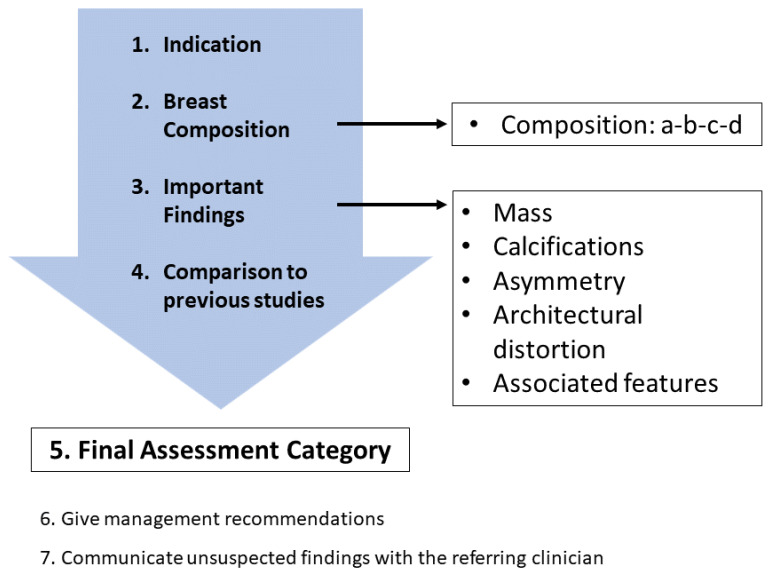
Standard reporting system.

Mammography databases can offer various types of information. To better understand this information, it is valuable to understand the image examination process of a radiologist. The standard mammographic examination consists of four images, two projections for each breast: the craniocaudal (CC) view, which is a top-to-bottom view, and the mediolateral oblique (MLO) view, which is an oblique view (Figure 1). The ACR BI-RADS Atlas 2013 [10] was designed to standardize breast imaging reporting (Figure 2).

**Figure 3:**
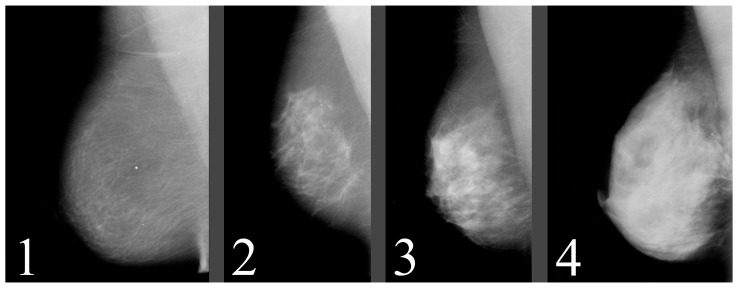
ACR standardized breast density. Source: [12].

For our purposes, breast composition is the first important step. In the older BI-RADS edition, the assignment was based on the four ACR (American College of Radiology) categories, which were based on the density of fibroglandular tissue in the breast (Figure 3, Table 1). However, in the 2013 edition, the standard was changed to categories a–d (Table 2), which consider the likelihood that a mass may be obscured by fibroglandular tissue.

Important findings are the next step in the image annotation process. The most common abnormalities found in mammography are masses, calcifications, architectural distortions, and asymmetries. Fortunately, benign abnormalities are much more common than malignant ones, with studies showing that 60% to 80% of all breast lumps are benign [13].

**Figure 4:**
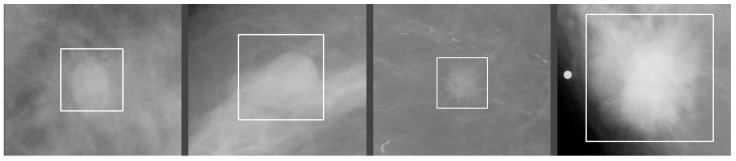
Different types and margins of masses. Source: [12].

**Figure 5:**
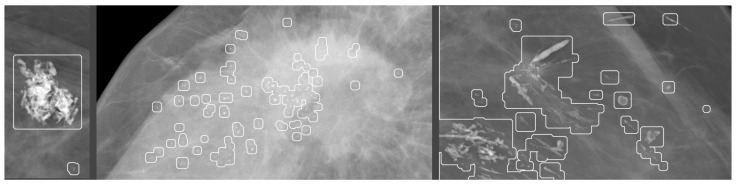
Different types of calcifications. Source: [11].

**Figure 6:**
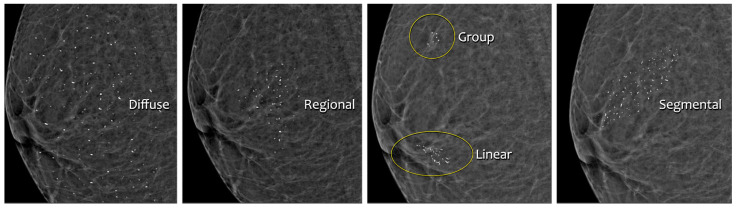
Distribution of calcifications. Source: [17].

**Figure 7:**
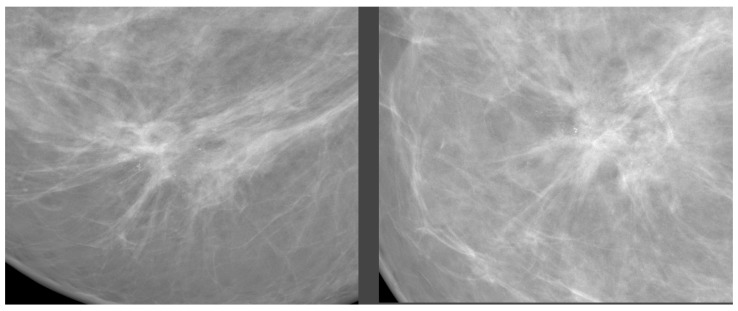
Architectural distortion examples. Source: [11].

**Figure 8:**
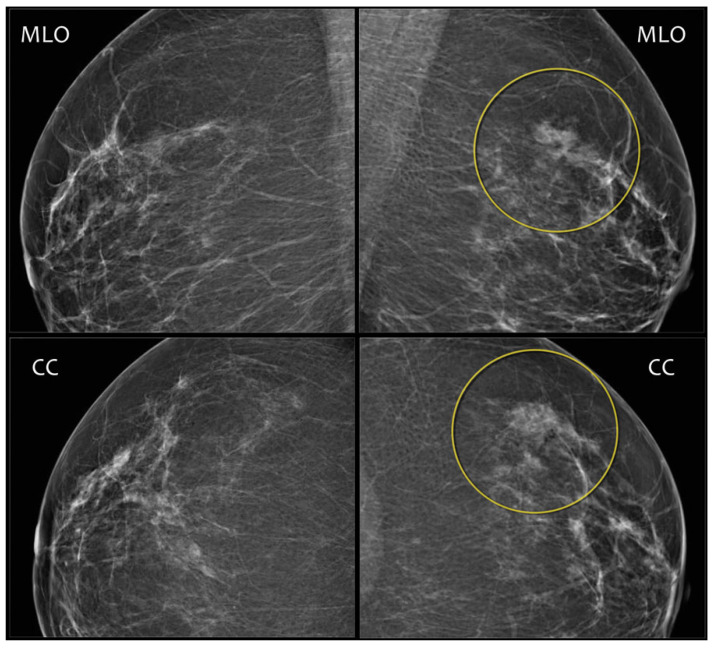
Asymmetry seen on LMLO and LCC views. Source: [17].

- Masses are three-dimensional space-occupying lesions in the breasts [14], Figure 4. A mass must be visible in both projections and demonstrate partially or completely convex-outward borders. Masses can vary in shape, margins, and density.

- Calcifications are deposits of calcium salts in the breast [15], Figure 5. They are present in approximately 85% of mammograms and can vary in morphology and distribution, see Figure 6.
  – Diffuse: Calcifications are randomly distributed throughout the breast. In most cases, this is a benign finding.
  – Regional: Calcifications occupy a significant proportion of breast tissue with the greatest dimension of more than 2 cm. It is expected that calcifications are not in the milk ducts, and therefore benignity is more likely.
  – Group (Cluster): At least five calcifications occupying a small portion of breast tissue (1–2 cm). It can be a benign or malignant finding. If only one group is present in the breast, the probability of malignancy is increased.
  – Linear: Calcifications are arranged in a line, corresponding to their location in the ducts, and therefore indicating the malignancy of the finding.
  – Segmental: Calcifications suggest deposits in a duct or ducts and their branches, indicating malignancy. Differentiating between regional and segmental calcifications can be problematic.
  Of the breast cancers detected on mammography due to calcifications, about two-thirds represent DCIS (ductal carcinoma in situ). DCIS is a noninvasive or pre-invasive type of breast cancer, which means that calcifications can lead to cancer detection in the earliest stage. Approximately 95% of all DCIS cases are diagnosed based on mammographically detected microcalcifications [16].

- Architectural distortion (Figure 7) is a term used when the normal architecture of the breast is distorted without a definite mass being visible [17]. This can include thin straight lines or spiculations radiating from a point, as well as focal retraction, distortion, or straightening at the edges of the parenchyma.

- Asymmetries represent a range of morphological descriptors for unilateral fibroglandular density findings seen on one or more mammographic projections that do not meet the criteria for a mass [18], as shown in Figure 8. For example, if the three-dimensionality of a mass is unconfirmed, then the finding should be marked as an asymmetry.

After annotating abnormalities, radiologists compare the latest images with images from previous studies, searching for dynamic changes in masses, calcifications, and other abnormal findings. Additionally, the volume of fibroglandular tissue decreases with age, which may uncover previously unseen findings hidden behind the tissue.

The last important step is the BI-RADS assessment, Table 3. Every examination of images should result in one of seven BI-RADS assessment categories. If the final category is 4 or 5, then a tissue biopsy for a definitive diagnosis is required. The biopsy result holds the most objective information about the malignancy of the suspicious finding.

## 3. Open-Access Databases

**Figure 9:**
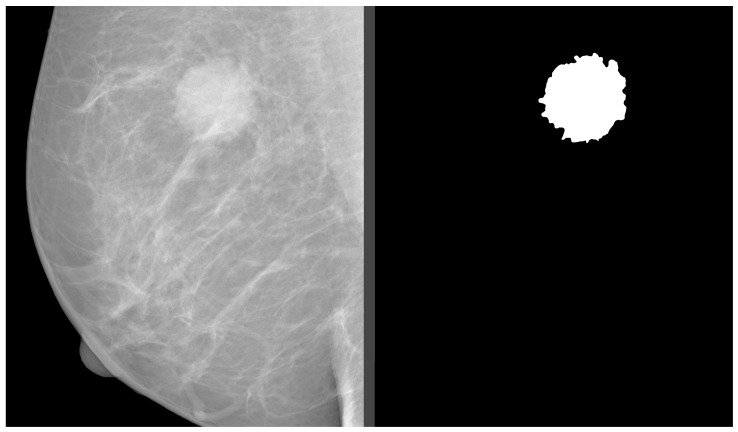
Binary mask of mass finding. Source: [11].

**Figure 10:**
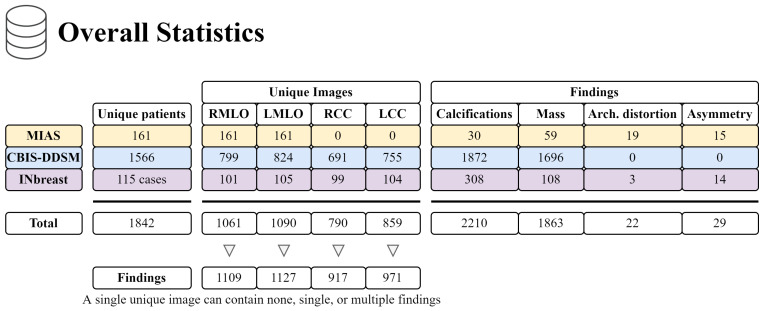
Overview of the MIAS, CBIS-DDSM, and INbreast datasets.

**Figure 11:**
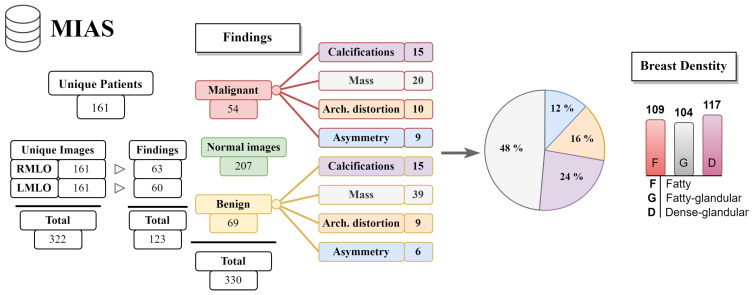
The MIAS database statistics.

**Figure 12:**
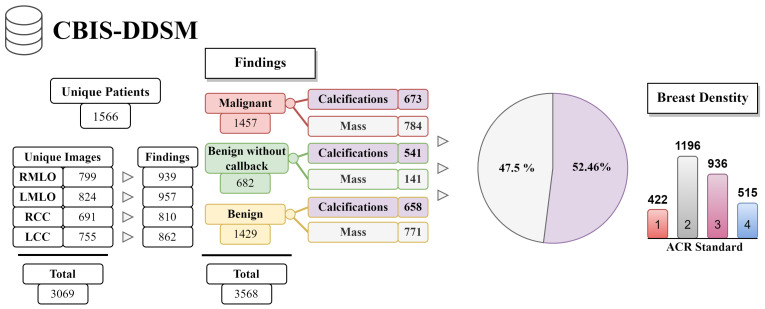
The CBIS-DDSM database statistics.

**Figure 13:**
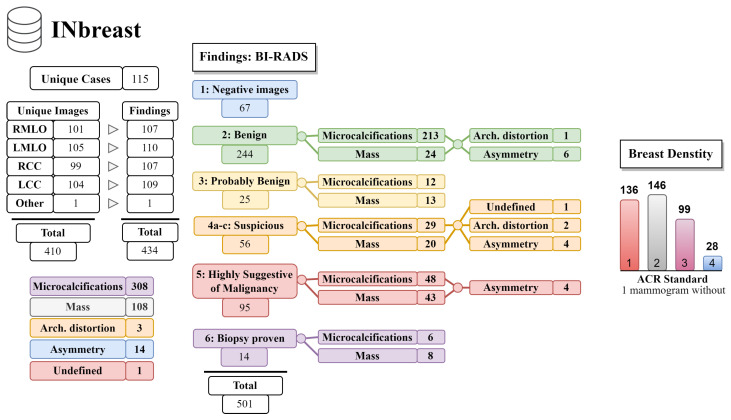
The INbreast database statistics.

In our study, we analyzed mammography databases that contained information about the boundaries and malignancy of findings. The region of interest (ROI) can be defined in several ways, with the most common method being a binary mask (as shown in Figure 9). The mask is an image with the same resolution as the observed image, where pixels have only two values. The first value (usually 0) represents the background, while the second value (usually 1 or 255) represents the ROI. Alternatively, the ROI can be defined by the coordinates of the center with the radius of a circle around the ROI, or by contours around the ROI. These databases are of significant value for neural network models, as training with whole images would be more complex and time-consuming. Even databases with only image-level labels have value in deep learning, for example, they can be used for fine-tuning a model. A summarized overview of the analyzed datasets (excluding the OMI-DB, as the amount of data may vary) is shown in Figure 10 and Table 4, while more detailed statistics of the individual datasets are provided in Figure 11, Figure 12 and Figure 13.

### 3.1. The Mammographic Image Analysis Society Digital Mammogram Database (MIAS)

The dataset [19] was released in 1994 and can be accessed at http://peipa.essex.ac.uk/info/mias.html (accessed on 10 March 2022) without any registration requirement. Each finding in the dataset includes details about the breast density, the abnormality type, the severity of the abnormality, the x and y image coordinates of the center of the abnormality, and the approximate radius of a circle that encloses the abnormality. The coordinate system origin is located in the bottom-left corner. In cases where calcifications are diffusely distributed instead of being concentrated in a single area, center locations and radii are not applicable and have been omitted. Furthermore, center locations and radii are generally applied to clusters rather than individual calcifications.

Each image abnormality is classified into one of seven categories, including calcification, well-defined/circumscribed masses, spiculated masses, other ill-defined masses, architectural distortion, asymmetry, and normal. The database comprises only images in the MLO view, with each patient having both a left MLO (LMLO) and a right MLO (RMLO) view, stored in Portable Gray Map (PGM) format.

**Figure 14:**
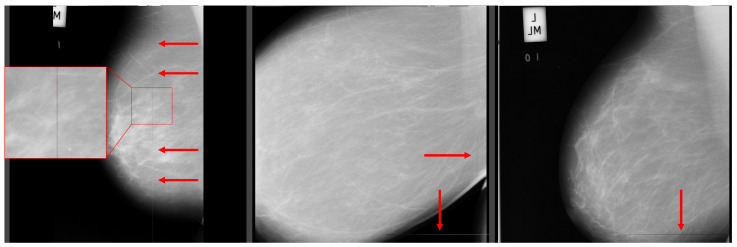
Artifacts pointed to by arrows, left and middle images without pectoral muscle, and middle and right images do not contain whole breasts. Source: [19].

**Figure 15:**
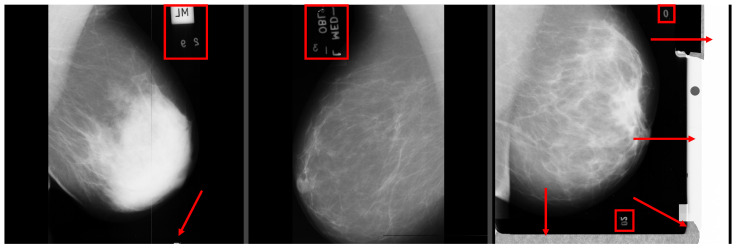
Redundant elements marked with red squares and arrows. Source: [19].

One disadvantage of the dataset is the low resolution of the images, which is only 1024 × 1024 pixels. This low resolution may potentially result in a loss of important information about microcalcifications. Moreover, many mammograms in the dataset may contain breast positional mistakes [20] and artifacts [21] (as shown in Figure 14). Additionally, the breast density assessment in the dataset does not meet the standards of the ACR BI-RADS Atlas. Since the images were acquired using the digitalization of the SFM method, they may contain unnecessary elements (as shown in Figure 15). These elements could have a negative impact during neural network training, and therefore, their removal should be considered.

The dataset has been used in various studies, such as the work of Ragab et al. [22], where the authors presented a CAD (computer-aided diagnosis) system. They employed a combination of DCNN (deep convolutional neural network) and SVM (support vector machine), where the DCNN was used for deep feature extraction, which was then fed into an SVM classifier with different kernel functions. The classifier was used to determine whether the input should be classified as normal or abnormal. Using this approach, they achieved an accuracy of 95.4% on the MIAS dataset. However, when using only the DCNN for classification, they achieved an accuracy of only 74.4% with the best model. Similarly, the work of Pillai et al. [23] also utilized the MIAS dataset to experiment with different convolutional architectures. In their research, the VGG16 architecture stood out the most, achieving an accuracy of 75.46% in determining whether a whole mammogram belongs to the normal or abnormal category.

### 3.2. Curated Breast Imaging Subset of Digital Database for Screening Mammography (CBIS-DDSM)

The CBIS-DDSM [12] is an updated and standardized version of the DDSM. The subset has updated ROI segmentation, and the bounding boxes and images have been decompressed and converted to DICOM (Digital Imaging and Communications in Medicine) format, which is a standardized format for all medical images. The original DDSM database was released in 1997. The dataset can be freely obtained at https://wiki.cancerimagingarchive.net/pages/viewpage.action?pageId=22516629 (accessed on 10 March 2022). To download the data, an NBIA Data Retriever is required, which is specialized software for opening the TCIA (The Cancer Imaging Archive) manifest files from the website.

The CBIS-DDSM dataset is divided into two groups based on the type of abnormality, which are calcifications and masses. Each abnormality group has a training and a testing set. The dataset provides additional details for each abnormal finding, including the breast density standardized by ACR (American College of Radiology), BI-RADS (Breast Imaging Reporting and Data System) assessment, the pathology result, and the rating of the subtlety of the abnormality. For calcification findings, information about their type and distribution is included, while for mass findings, information about their shape and margins is provided. The ROI (Region of Interest) is defined by the binary mask. The images in the CBIS-DDSM dataset are in high resolution, with slight variations in resolution among the images.

Processing the CBIS-DDSM dataset presented several challenges. Some of the difficulties encountered include:

1. Mirrored images: Some images in the dataset were mirrored, where the breast positioning in the right medio-lateral oblique (MLO) image appeared to be the same as that in the left MLO image, and vice versa.
2. Inconsistent filenames: The filenames of the images did not always correspond with the filenames in the accompanying description CSV file.
3. Resolution discrepancies: Some masks in the dataset had a different resolution than the original image. This required rescaling of the masks to match the resolution of the corresponding images, ensuring the proper alignment and accurate localization of abnormalities.
4. Oversized calcification masks: Some calcification masks in the dataset were oversized and did not accurately indicate the presence of calcifications in the corresponding areas of the images.
5. Redundant elements in images: Similar to the MIAS database, images in the CBIS-DDSM dataset may contain redundant elements due to the acquisition with the SFM (scan-film mammography) technology.
6. Artifacts in mammograms: Some mammograms in the dataset may contain artifacts, which could affect the accuracy and reliability of the analysis.

These challenges highlight the importance of careful preprocessing and data cleaning steps in working with medical image datasets, to ensure accurate and reliable results in subsequent analysis and modeling tasks.

The CBIS-DDSM or DDSM database has been utilized in numerous studies. One notable example is the work by Shen et al. [24], where they employed an end-to-end training approach. This approach involved initially training the model on image patches with findings, and subsequently converting the classifier to allow fine-tuning on entire images that did not contain Regions of Interest (ROIs) but only labels indicating the presence or absence of malignant tissue. The best-performing model achieved an AUC (Area Under the ROC Curve) of 0.88 on the dataset. When averaging results from four models, an AUC of 0.91 was achieved, with a sensitivity of 86.1% and a specificity of 80.1%.

In another publication by Ragab et al. [22], which employed a combination of deep features and an SVM classifier, the CBIS-DDSM dataset was also used in addition to the MIAS dataset. Experiments on the CBIS-DDSM dataset were considered more realistic as it contains a larger amount of data, but the authors focused solely on masses. The masses were classified into benign or malignant classes. By using a combination of DCNN and SVM, they achieved the highest accuracy of 93.7%, while using only the DCNN classifier resulted in an accuracy of 76.01%.

### 3.3. INbreast

**Figure 16:**
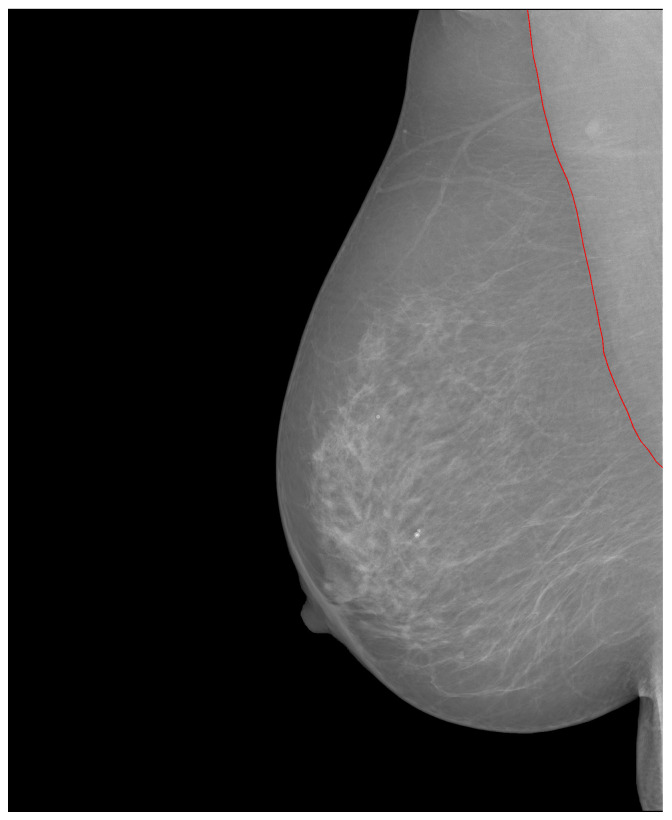
Lined contour points of the pectoral muscle. Source: [11].

At the time of writing, the INbreast database [11] was no longer available on the official website. Mammograms in this database are stored in the DICOM format, with a resolution of either 4084 × 3328 or 3328 × 2560, depending on the breast size. The database includes images with various abnormalities such as microcalcifications, masses, architectural distortions, and asymmetries. Regions of Interest (ROIs) are defined by contour points in an XML (extensible markup language) file. Contour annotation of the pectoral muscle is also included (refer to Figure 16). Additionally, there is an extra folder that provides masks for calcifications and masses. However, it is worth noting that there are only 130 calcification masks out of 308 images with calcifications, and 107 mass masks out of 108 images with mass findings.

One advantage of the dataset is that calcifications are described by individual contours instead of the contours of their area, providing more precise localization information. Furthermore, mammograms in the dataset were obtained using FFDM technology, which avoids redundant visual information such as view and laterality. However, it is worth noting that the database does not provide any histopathological results for the findings, and only BI-RADS assessment is available. The absence of histopathological results is likely the greatest limitation of the dataset.

The usage of the dataset can be observed in the work of Singh et al. [25], which focused on generating realistic binary masks using cGAN (conditional generative adversarial networks). The authors were able to achieve a high Dice coefficient of 94% and an Intersection over Union of 87%. Another study by Shen et al. [24] also utilized the dataset, along with the CBIS-DDSM database, as an independent test set. In this study, the best model achieved an AUC of 0.95 on the INbreast database.

### 3.4. OPTIMAM Medical Image Database (OMI-DB)

The OMI-DB database [26] stands out from previous databases as it continues to grow in size every year. It collects images and information from several screening centers across the UK, including images without any suspicious findings. Due to the complex structure of the database, it is recommended to use the “omidb” Python package to extract and process information from it.

While the database is publicly available, there are certain limitations. Access to the database requires justification of the research project, and the applicant must have a relevant research history and affiliation with a healthcare institution, academic center, nonprofit organization, or commercial organization. Further information on how to gain access can be found at https://medphys.royalsurrey.nhs.uk/omidb/getting-access/ (accessed on 10 December 2022). Once the project is approved, the applicant will not have access to the entire dataset, but will need to specify the data they prefer to work with, such as images containing a certain type of finding.

The OMI-DB database [26] currently contains data from 179,326 patients (Table 5). It is important to note that one patient may have multiple malignant or benign episodes. The malignancy of a finding is determined by biopsy. The images in the database are stored in DICOM format, and for each patient, there may be ROI information for multiple views (e.g., MLO + CC) described with bounding rectangles. Each rectangle carries information about the type of finding, and multiple findings (e.g., mass + calcifications + architectural distortion) may be present in a single rectangle. The most common findings in the database are masses and suspicious calcifications.

One significant advantage of the OMI-DB database is that it includes previous mammograms from screening exams, taken before the finding had developed or was detected. This provides an opportunity to study the evolution of findings over time. Other benefits of the database include the large amount of available data and increased presence of rare findings such as architectural distortions and asymmetries. However, a disadvantage of the database is its complexity, as it may require additional time for processing due to exceptions and border cases.

An example of an article that documents the use of the OMI-DB dataset is the work by Cantone et al. [27], which compares Deep Convolutional Neural Networks (DCNN) with Vision Transformers (ViT). The research showed that some ViT architectures can achieve comparable results to traditional DCNN architectures. Overall, the best-performing model was found to be a DCNN with the modern EfficientNet architecture, which achieved an accuracy of up to 85.2% (sensitivity: 74.5%, specificity: 91.5%).

## 4. Conclusions

In the first part of our article, we provided a detailed description of the mammography assessment process to provide insights into the information provided by open-access databases. The second part of our article included a comprehensive description and analysis of four databases: INbreast, MIAS, CBIS-DDSM, and OMI-DB. We also discussed the advantages and disadvantages of each database.

Despite the limitations of these datasets, such as artifacts, positional mistakes, mirrored images, or incorrect filenames, they are still suitable for solving various machine learning tasks, such as the classification or detection of abnormal tissue. However, image preprocessing techniques can be employed to address these issues.

Moreover, recent studies have demonstrated the usability of these databases by implementing Deep Convolutional Neural Networks (DCNN) and other machine learning models to improve the accuracy of breast cancer diagnosis. In the future, these models have the potential to reduce the time-consuming nature of examinations by replacing the need for a second radiologist in double reading.

The combined total of unique images from the described databases, excluding the OMI-DB due to the variations in data depending on the agreement, was 3801, with 4125 annotated findings.

The vast majority of these images come from the CBIS-DDSM database. However, this database contains multiple errors; so, careful preprocessing is necessary. The database is suitable for solving a variety of classification tasks. Its status as the most widely used database and its inclusion of a data split into training and testing sets facilitates the comparison of results with those from other studies.

The INbreast seems to have images of higher quality; however, they lack histopathological conclusions. Given that the database is the only one containing individually described calcifications, it is well-suited for addressing various machine learning detection tasks.

The OMI-DB is by far the largest database containing high-quality images, but to access them, one needs to undergo a long process of project writing, evaluation, and agreement signing. A significant advantage of the database is the presence of images from the patient’s previous examinations, which can be used to observe dynamic changes in the breasts. The use of such mammographic data in machine learning is relatively new and unexplored.

The MIAS database was found to be the least suitable for machine learning tasks, mainly due to its non-standard low resolution and limited number of images. Therefore, we recommend using it primarily for validation purposes to confirm the effectiveness of models trained on other datasets.

The main challenge in training deep neural networks is the relatively small size of these datasets. However, with appropriate techniques, reasonable results can still be achieved even with small-sized datasets.

## Figures and Tables

**Table 1:** ACR standardized categories.

Category	Fibroglandular Tissue
1	<25%
2	25–50%
3	50–75%
4	>75%

**Table 2:** New categories from ACR BI-RADS Atlas 2013.

Category	Description
a	The breasts are almost entirely fatty
b	There are scattered areas of fibroglandular density
c	The breasts are heterogeneously dense, which may obscure small masses
d	The breasts are extremely dense, which lowers the sensitivity of mammography

**Table 3:** BI-RADS assessment categories.

**Category 0**	**Mammography:** Incomplete—Need Additional Imaging Evaluation and/or Prior Mammogram Comparison		
	**Ultrasound and MRI:** Incomplete—Need Additional Imaging Evaluation		
**Category 1**	Negative		
**Category 2**	Benign		
**Category 3**	Probably Benign		
**Category 4**	Suspicious	Mammography and Ultrasound	Category 4A: Low suspicion of malignancy
			Category 4B: Moderate suspicion of malignancy
			Category 4C: High suspicion of malignancy
**Category 5**	Highly Suggestive of Malignancy		
**Category 6**	Known Biopsy-Proven Malignancy		

**Table 4:** Specific features of the databases with appropriate neural network tasks.

Database	Image Format	Abnormality Types	BI-RADS	Breast Density	Histopathology	Ground Truth	Imaging Modality	Image Resolution	Tasks
MIAS	PGM	calcifications, masses, architectural distortion, asymmetries	no	non-standard categories	no	circle	SFM	1024 × 1024 (low)	classification, detection
CBIS-DDSM	DICOM	calcifications, masses	yes	old ACR	yes	binary mask	SFM	high	classification, detection, segmentation
INbreast	DICOM	calcifications, masses, architectural distortions, asymmetries	yes	old ACR	no	contour points or binary mask	FFDM	4084 × 3328 or 3328 × 2560 (high)	classification, detection, segmentation
OMI-DB	DICOM	calcifications, masses, architectural distortion, asymmetries	no	no	yes	rectangle (may contain a combination of abnormalities)	FFDM	high	classification, detection

**Table 5:** A summary of the number of clients hosted in the database with the specified case.

Category	Count
Normal	166,694
Benign	4055
Malignant	7417
Interval Cancer	1160

## Data Availability

Analyzed databases: MIAS [19]—http://peipa.essex.ac.uk/info/mias.html (accessed on 10 March 2022); CBIS-DDSM [12]—https://wiki.cancerimagingarchive.net/pages/viewpage.action?pageId=22516629 (accessed on 10 March 2022); INbreast [11]—database is no longer available from original source; OMIDB [26]—https://medphys.royalsurrey.nhs.uk/omidb/getting-access/ (accessed on 10 December 2022).
